# Non-Lethal Heat Shock Increased Hsp70 and Immune Protein Transcripts but Not Vibrio Tolerance in the White-Leg Shrimp

**DOI:** 10.1371/journal.pone.0073199

**Published:** 2013-09-09

**Authors:** Nguyen Hong Loc, Thomas H. MacRae, Najiah Musa, Muhd Danish Daniel Bin Abdullah, Mohd. Effendy Abdul Wahid, Yeong Yik Sung

**Affiliations:** 1 Department of Aquaculture Science, Faculty of Fisheries and Aqua-Industry, Universiti Malaysia Terengganu, Kuala Terengganu, Malaysia; 2 Department of Biology, Dalhousie University, Halifax, Nova Scotia, Canada; 3 Department of Fisheries Science, Faculty of Fisheries and Aqua-Industry, Universiti Malaysia Terengganu, Kuala Terengganu, Malaysia; 4 Institute of Marine Biotechnology, Universiti Malaysia Terengganu, Kuala Terengganu, Malaysia; National Science and Technology Development Agency, Thailand

## Abstract

Non-lethal heat shock boosts bacterial and viral disease tolerance in shrimp, possibly due to increases in endogenous heat shock protein 70 (Hsp70) and/or immune proteins. To further understand the mechanisms protecting shrimp against infection, Hsp70 and the mRNAs encoding the immune-related proteins prophenoloxidase (proPO), peroxinectin, penaeidin, crustin and hemocyanin were studied in post-larvae of the white-leg shrimp *Litopenaeus vannamei,* following a non-lethal heat shock. As indicated by RT-qPCR, a 30 min abrupt heat shock increased Hsp70 mRNA in comparison to non-heated animals. Immunoprobing of western blots and quantification by ELISA revealed that Hsp70 production after heat shock was correlated with enhanced Hsp70 mRNA. proPO and hemocyanin mRNA levels were augmented, whereas peroxinectin and crustin mRNA levels were unchanged following non-lethal heat shock. Penaeidin mRNA was decreased by all heat shock treatments. Thirty min abrupt heat shock failed to improve survival of post-larvae in a standardized challenge test with *Vibrio harveyi*, indicating that under the conditions of this study, *L. vannamei* tolerance to Vibrio infection was influenced neither by Hsp70 accumulation nor the changes in the immune-related proteins, observations dissimilar to other shrimp species examined.

## Introduction

Exposure to long-term hyperthermic stress disrupts the normal physiological processes of shrimp and under severe conditions may decrease feeding, retard growth and molting, and weaken the immune response, resulting in mortality [1]. However, a non-lethal heat shock (NLHS) enhances shrimp resistance against bacterial and viral infections. For example, heating from 28°C to 37°C for 30 min with 6 h recovery shielded the brine shrimp *Artemia* against *Vibrio campbellii* and *V. proteolyticus* challenge, with survival enhanced 2-fold over non-heated animals [2]. A NLHS from 29°C to 35°C for 24 h increased the tolerance of the Tiger shrimp *Penaeus monodon* to gill associated virus (GAV) by reducing viral replication [3].

Several mechanisms have been proposed to explain how heat shock defends against bacterial and viral diseases in aquatic animals. A NLHS may enhance heat shock protein (Hsp) production, particularly Hsp70, which is required to stabilize cells against injury due to pathogen proliferation, properly fold cell proteins synthesized in response to bacterial pathogens, store and re-fold partially denatured proteins and stimulate the innate immune response [4]. On the other hand, NLHS may increase the expression of selected immune-related genes resulting in enhanced immunity. In this context, heating from 24°C to 27°C and 30°C stimulates the prophenoloxidase (proPO) cascade system of *L. vannamei*, a mechanism important for pathogen melanisation by the innate immune system [5]. proPO occurs naturally as an inactive pro-form of phenoloxidase (PO), activated by an endogenous trypsin-like serine protease referred to as prophenoloxidase activating enzyme (ppA) [6]. proPO activates melanin production and increases cell adhesion, encapsulation and phagocytosis [7], [8], important mechanisms by which the crustacean innate immune system combats microbial invasion.

An associated protein of the proPO system, referred to as peroxinectin, mediates cellular adhesion to invading microorganisms and enhances the antimicrobial oxidase burst ability of hemocytes [9]. This protein is a secreted opsonin [10] which regulates granule exocytosis and promotes encapsulation of foreign particles [11]. Peroxinectin normally occurs in the granular and semigranular hemocytes of *L. vannamei.* Elevated synthesis of transcripts in the gills indicates peroxinectin traps and encapsulates invasive bacteria in this organ, a putative defense mechanism for pathogen elimination in shrimp [12]. Penaeidins are the most prominent antimicrobial peptides in shrimp, occurring naturally in the hemolymph. Five types of penaeidins exist of which three, namely penaeidin-1, -2, -3 were isolated from hemocytes of *L. vannamei* [13]. Pen-4 and Pen-5 were isolated from hemocyte organelle-rich fractions of *L. vannamei* [14] and *P. monodon* [15]. Penaeidins possess potent antifungal and antibacterial properties [16] and they are effective against filamentous fungi and Gram positive bacteria [17]. However, the penaeidins function weakly against yeast such as *Candida albican* and *Sacharomyces cerevisiae* and Gram negative bacteria, including *Vibrio sp* [18]. Of the five penaeidins, Pen-3 is the most effective and it is abundant in the hemocytes of *L. vannamei* [13] and *P. monodon* [19]. Hemocyanin, an oxygen transporter unique to crustaceans [20], plays a crucial role in protein storage, osmotic regulation [21] and ecdysone transportation [22]. Astacidin, a hemocyanin from crayfish, exhibits anti-bacterial activities [23] and hemocyanins isolated from *L. vannamei* and *P. stylirostris* have antifungal capabilities [24]. Hemocynin from *P. monodon* possesses non-specific antiviral properties [25]. Crustins are cationic cysteine-rich antimicrobial peptides consisting of a single whey acidic protein (WAP) domain recognized as a signature motif for a serine protease inhibitor [26]. Two types of crustins, namely crustin II and III, are abundant in the hemocytes of *P. monodon*, each having different sequence characteristics and antimicrobial activities [27]. Most crustins exhibit potent activity against Gram positive bacteria [28] but are weak against Gram negative bacteria and fungi [29], except the crus-like*Pm* which kills some Gram negative bacteria [30]. Certain isoforms of crustin have biological functions other than immunity. Crustin*Pm5*, a type II crustin gene with complete heat shock regulatory elements (HSEs), upregulates robustly under heat and salinity stress, suggesting involvement in abiotic stress tolerance of the Penaeid shrimp [31].

Because the relationship between NLHS and the innate immune response is poorly understood, the formulation of strategies that favor shrimp tolerance to bacterial infection, and other important diseases in aquaculture is hampered. The effect of NLHS on the production of Hsp70 and of mRNAs encoding the immune proteins, proPO, peroxinectin, penaeidin, crustin and hemocyanin in *L. vannamei* post-larvae, an intermediate stage between mysis and juveniles in the Penaeid shrimp life cycle, was studied. The synthesis of Hsp70 mRNA and protein were induced by NLHS. The production of mRNAs encoding proPO and hemocyanin was enhanced whereas penaeidin mRNA was reduced. This study is the first to demonstrate that NLHS has a differential effect on mRNAs encoding proteins involved in the innate immune response of *L. vannamei*. Although the mRNA for the immune proteins proPO and hemocyanin increased, along with Hsp70 upon NLHS, *L. vannamei* post-larvae did not acquire increased tolerance to infection.

## Methodology

### Heat Shock of *L. vannamei*

Post-larvae of *L. vannamei* were acclimated at 28°C, 30 g/L salinity and 50 animals/L stocking density for 7 days prior to use. During acclimation, animals were fed twice daily to satiation with live *Artemia* nauplii. Faeces were withdrawn and 50% of the rearing water was replaced daily. Gentle aeration maintained dissolved oxygen (DO) at 5 ppm. Post-larvae were exposed to abrupt 30 min heat shocks from 28°C to 30°C, 32°C, 34°C, 36°C and 38°C with an immersion circulator water bath system (WiseCirCu®, Germany) accurate to ±0.5°C. Post-larvae were transferred immediately after heating to 28°C for 8 h recovery prior to sampling of protein and RNA. Control post-larvae were maintained at 28°C.

### Quantification of mRNAs by RT-qPCR

Total RNA was extracted from 100 mg of *L. vannamei* post-larvae with TRIsure™ following manufacturer’s instructions (Bioline, U.K.). The purity and quantity of the RNA were determined spectrophotometrically at 260 and 280 nm. First strand cDNA was obtained from 1 µg total RNA with a cDNA synthesis kit (Bioline, UK) by incubating 45 min at 42°C following manufacturer’s instructions. PCRs were done in the absence of reverse transcript to confirm the lack of DNA carryover.

RT-qPCR was performed with 2×SensiMix SYBR No-ROX kit (Bioline, UK) with forward and reverse primers specific for proPO, peroxinectin, penaeidin, crustin, haemocyanin, Hsp70 and β-Actin (Table 1). Amplification was in a Miniopticon Real-time PCR system (Bio-Rad, USA) at 95°C for 10 min followed by 40 cycles of 95°C for 15 s and 58°C for 1 min for immune-related mRNA or 40 cycles of 95°C for 5s and 60°C for 31s for Hsp70. Melting curve analysis of PCR products was performed to confirm that only one product was amplified. The cycle threshold (C_T_) values were recorded by Opticon Monitor 3 software (Bio-Rad, USA) and fold difference in quantity for each immune-related cDNA, relative to the β-actin gene, was calculated by the 2^−ΔΔCt^ method [32]. Amplifications were done with 3 replicates for each heat shock treatment and samples were collected from two separate experiments.

**Table 1:** Accession numbers and primer sequences for RT-qPCR.

Gene	Accession #	Primer sequence (5′–3′)Forward (F) and Reverse (R)	Size bp	References
Hemocyanin	X82502	(F) TCTTAGTGGTTCTTGGGCTTGTC(R) GGTCTCCGTCCTGAATGTCTCC	124	[43]
Crustin	AF430076	(F) ACGAGGCAACCATGAAGG(R) AACCACCACCAACACCTAC	141	[43]
Penaeidin-3a	Y14926	(F) CACCCTTCGTGAGACCTTTG(R) AATATCCCTTTCCCACGTGAC	121	[43]
Peroxinectin	AF188840	(F) CGAAGCTTCTTGCAACTACCA(R) GCAGGCTGATTAAACTGGCTT	56	[37]
Prophenoloxidase	AY723296	(F) CGGTGACAAAGTTCCTCTTC(R) GCAGGTCGCCGTAGTAAG	122	[43]
Hsp70	EF495128	(F) CCTCCAGGACTTCTTCAACG(R) GGTCACGTCCAACAGCAAC	144	[50]
β- actin	AF300705	(F) CCACGAGACCACCTACAAC(R) AGCGAGGGCAGTGATTTC	142	[43]

### Immunodetection of Hsp70

Protein extraction was performed as described with minor modifications [33]. Fifty mg of post-larvae was homogenized in 500 µl cold buffer K (150 mM sorbitol, 70 mM potassium gluconate, 5 mM MgCl_2_, 5 mM NaH_2_PO_4_, 40 mM HEPES, pH 7.4) [34] containing protease inhibitor cocktail (Catalogue #P8340, Sigma-Aldrich, Missouri, USA) at the highest recommended level. After centrifugation at 4000×*g* for 3 min at 4°C, 10 µl aliquots of supernatant were combined separately with 5 µl SDS sample buffer, mixed and heated at 95°C for 5 min. Samples were cooled and centrifuged at 2200×*g* for 1 min. Fifty µg of protein sample was loaded in each lane of 10% SDS polyacrylamide gels and electrophoresis was at 120 V for 15 min followed by 150 V for 45 min [2]. Gels were either stained with Coomassie Biosafe (BioRad, USA) or blotted to polyvinylidene fluoride transfer membrane (BioRad Immun-Blot™ PVDF, USA) for probing with antibodies. Membranes were incubated with blocking buffer (50 ml of phosphate buffered saline containing 0.2% (v/v) Tween-20 and 5% (w/v) bovine serum albumin) at 25°C for 60 min at room temperature and then with a mouse monoclonal antibody specific to Hsp70 (Product# MA3-006) (Pierce Biotechnology, Rockford, USA), diluted 1∶5000. Membranes were washed 3 times for 5 min with Tris-buffered saline Tween-20 prior to incubation with HRP conjugated goat anti-mouse IgG (Pierce Biotechnology, Rockford, USA) diluted 1∶2500. Membranes were washed 3 times for 5 min with Tris-buffered saline Tween-20, and detection was with 0.7 mM diaminobenzidine tetrahydrochloride dehydrate in association with 0.01% (v/v) H_2_O_2_ in 0.1 M Tris-HCl, pH 7.6 [35].

### Hsp70 Quantification by ELISA

One hundred µl of MA3-006 antibody, diluted 1∶5000 in phosphate-buffered saline (PBS) was placed in each well of a 96-well round-bottom polystyrene plate (Nunc-Immunoplate Maxisorp, Denmark) and incubated 1 h at room temperature. The antibody was removed and the plates rinsed 3 times with PBS. One hundred µl blocking buffer (PBS containing 0.2% Tween-20 and 5% bovine serum albumin) was added to each well followed by incubation for 1 h at room temperature. The blocking buffer was decanted and the wells rinsed 3 times with PBS. One hundred µl of protein extract from *L. vannamei* diluted 10 times in PBS was added to each well and incubated 1 h at room temperature. The protein extracts were decanted and the wells rinsed 3 times with PBS prior to 1 h incubation at room temperature in 100 µl MA3-006 antibody diluted 1∶5000 in PBS. The antibody was discarded and the wells washed 3 times with PBS. One hundred µl of HRP conjugated goat anti-mouse IgG antibody (Affinity BioReagents Inc.) diluted 1∶5000 in PBS was added to the wells and incubated 1 h at room temperature. The wells were washed 3 times with PBS and incubated 30 min with 100 µl Tetramethylbenzidine (TMB) solution at room temperature. One hundred µl 1N H_2_SO_4_ was added to each well and after incubation for 30 min at room temperature, color intensity was determined at 450 nm in a microplate reader (Thermo Electron, USA). A standard curve, constructed with human Hsp70 recombinant protein (Sigma-Aldrich, USA) was used to convert sample absorbance to Hsp70 content with values expressed as human Hsp70 equivalent. All experiments were done in duplicate.

### Vibrio Culture

*V. harveyi* was grown at 28°C on Marine Agar 2216 (Difco Laboratories, Detroit, MI). Colonies were transferred individually to Marine Broth 2216 (Difco Laboratories) and grown to stationary phase by incubation overnight with constant shaking at 28°C. Bacteria were harvested by centrifugation at 1800×*g* for 10 min at 28°C, the supernatant removed and pellets suspended in filtered autoclaved sea water. Cell density was determined spectrophotometrically at 550 nm and the number of bacteria was calculated according to the McFarland standard (BioMerieux, Marcy L^’^Etoile, France) with an optical density of 1.0 corresponding to 1.2×10^9^ cells/ml [35].

### Development of a Vibrio Challenge Test System for *L. vannamei*

Thirty post-larvae reared at 28°C were incubated in separate aquaria containing 1L of filtered seawater with 1×10^1^, 1×10^3^, 1×10^5^ and 1×10^7^ *V. harveyi*/ml. Survival was determined 24, 36, 48 and 96 h after challenge by counting swimming animals. Each treatment was performed in triplicate and the survival percentage calculated as N_t_×100/N_o_ where N_t_ and N_o_ represent the final and initial number of post-larvae, respectively [35]. The lowest number of bacteria causing more than 50% mortality after 48 h was used for challenge tests. The experiments were repeated once.

### Cross-protection Experiment

Thirty post-larvae subjected to a NLHS from 28°C to 30°C, 32°C, 34°C, 36°C and 38°C, with a recovery period of 8 h, were challenged with 1.0×10^7^ *V. harveyi*/ml and their survival was determined after 24, 48 and 72 h. following methods just described. Moribund shrimp were counted as dead. Treatments were performed in triplicate and the experiments were repeated once.

## Results

### Quantification of mRNAs Encoding Immune-related Proteins after NLHS

**Figure 1:**
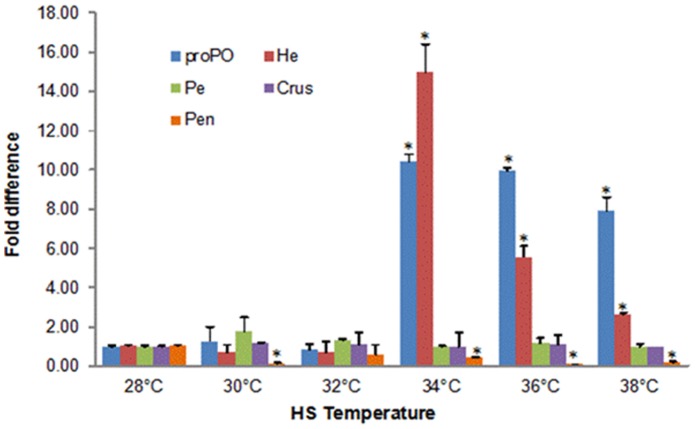
Quantification of mRNA encoding shrimp immune-related proteins following NLHS. Post-larvae were exposed to 30 min heat shock from 28°C to 30°C, 32°C, 34°C, 36°C and 38°C, then transferred to 28°C for 8 h. mRNAs encoding immune related proteins were quantified by RT-qPCR with beta-actin as reference. The error bars represent SD from 3 replicates. proPO, prophenoloxidase; He, hemocyanin; Pe, peroxinectin; Crus, crustin; Pen, penaedin; HS, heat shock. Asterisks denote statistically significant differences between values obtained for control and heat shocked post-larvae (*P<*0.05). The figure is a representation from two separate experiments.

When compared to non-heated post-larvae the mRNAs encoding proPO and hemocyanin increased significantly after NLHS with the former augmented approximately 10.0, 9.3 and 7.9-fold and the latter 15.0, 5.6 and 2.6-fold at 34°C, 36°C and 38°C respectively (Fig. 1). In both cases, the amounts of mRNA peaked at 34°C and declined at 36°C and 38°C. Peroxinectin and crustin mRNAs were unchanged by NLHS whereas penaeidin mRNA was reduced significantly (Fig. 1).

### Hsp70 mRNA and Protein Increased after NLHS

**Figure 2:**
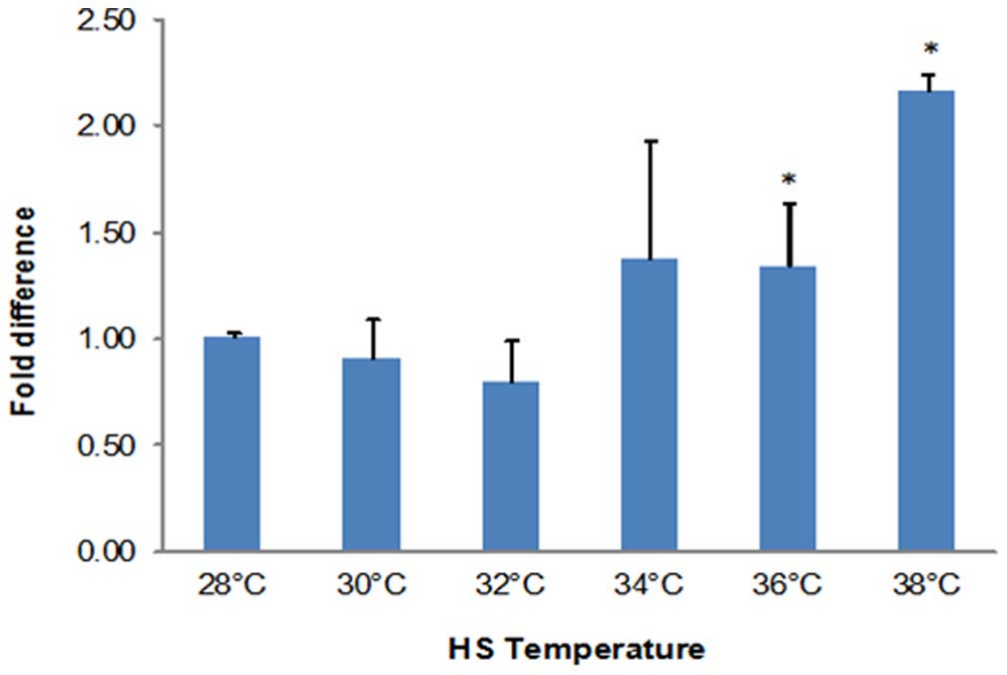
NLHS increased shrimp Hsp70 mRNA. Post-larvae were exposed to 30 min heat shock from 28°C to 30°C, 32°C, 34°C, 36°C and 38°C, then transferred to 28°C for 8 h. Hsp70 mRNA was quantified by RT-qPCR, with beta-actin as reference. Bars represent the fold difference of Hsp70 mRNA with comparison to the non-heated control. The error bars represent the SD from 3 replicates. Asterisks denote statistically significant differences between values obtained for control and heat shocked post-larvae (*P<*0.05). The figure is a representation from two separate experiments.

Thirty min NLHS from 28°C to 36°C and 38°C followed by recovery for 8 h increased Hsp70 mRNA approximately 1.4 and 2.1-fold, respectively (Fig. 2). Conversely, heating at 30°C and 32°C did not add to Hsp70 mRNA.

**Figure 3:**
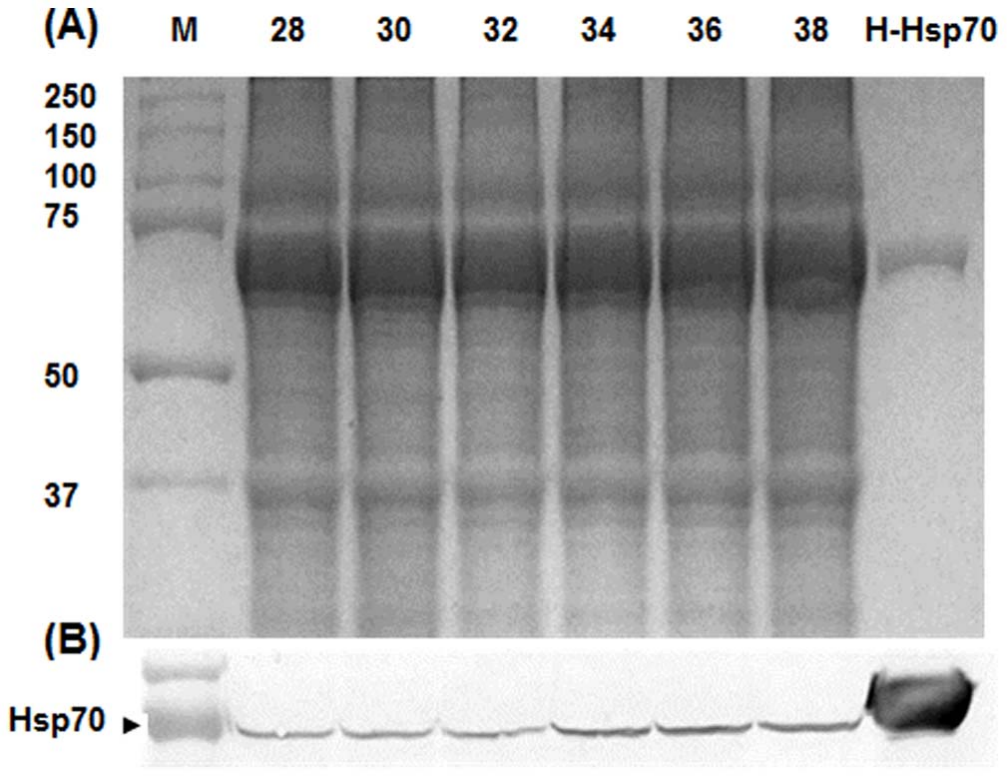
NLHS increased Hsp70 in *L. vannamei* post-larvae. Protein extracts were resolved by electrophoresis in SDS polyacrylamide gels and either stained with Coomassie Biosafe (A) or blotted to polyvinylidene fluoride membranes and incubated with antibody to Hsp70 (B). Approximately 50 µg of protein was loaded in each lane. 28, non-heat shocked post-larvae heat shock at 30°C, 32°C, 34°C, 36°C and 38°C was for 30 min with recovery at 28°C for 8 h H-Hsp70: Human Hsp70 recombinant protein M: molecular mass standards in kDa. The figure is a representation from two separate experiments.

**Figure 4:**
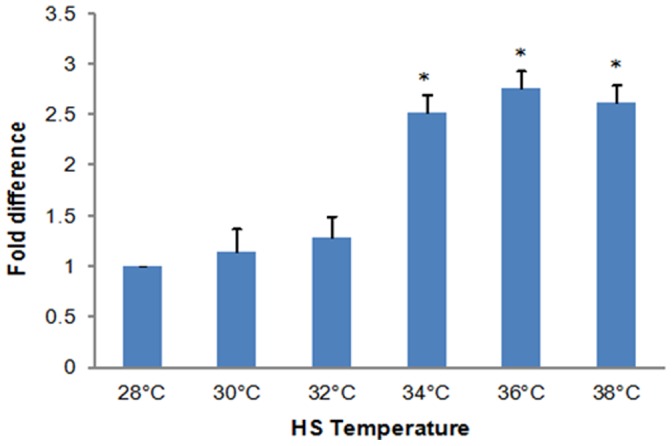
NLHS enhanced Hsp70 production in *L. vannamei*. Post-larvae were exposed to 30 min heat shock from 28°C to 30°C, 32°C, 34°C, 36°C and 38°C, then transferred to 28°C for 8 h. Quantification of Hsp70 was by ELISA. A standard curve, constructed with Human Hsp70 recombinant protein was used to convert ELISA readings obtained with shrimp tissue protein extracts to Hsp70 content. Bars represent the fold difference of Hsp70 quantity in comparison to a non-heated control. The error bars represent the SD from 3 replicates. Asterisks denote statistically significant differences between values obtained for control and heat shocked post-larvae (*P<*0.05). The figure is a representation from two separate experiments.

The protein extracts prepared from heated and non-heated shrimp were similar when resolved in SDS polyacrylamide gels and stained with Coomassie Blue (Fig. 3A). Immunoprobing of western blots revealed a single polypeptide of approximately 70 kDa which was boosted in amount by NLHS at 34°C and 38°C for 30 min (Fig. 3B). When compared to non-heated animals quantification by ELISA revealed that Hsp70 increased 2.5, 2.8 and 2.6-fold at the temperatures just mentioned (Fig. 4).

### Heat Shock had no Effect on Vibrio Tolerance

More than 50% mortality of *L. vannamei* post-larvae was caused by 1×10^7^ *V. harveyi*/ml in 48 h (Table 2) and these conditions were chosen for challenge testing. NLHS for 30 min at various temperatures followed by recovery for 8 h had no effect on the survival of *L. vannamei* post-larvae during *V. harveyi* challenge, with the number of surviving heat shocked animals not significantly different from non-heated animals (*P*>0.05) (Table 3).

**Table 2:** Survival (%) of *L. vannamei* post-larvae after 24, 48, 72 and 96 h challenges with 1×10^1^, 1×10^3^, 1×10^5^ and 1×10^7^ *V. harveyi*/mL.

Treatment	Dose (Bacteria/ml)	Survival (%)			
		24 h	48 h	72 h	96 h
Control	0	97±2^b^	86±2^c^	73±3^c^	61±5^b^
*V. harveyi*	1×10^1^	83±3^a^	66±2^b^	55±5^b^	33±3^a^
	1×10^3^	82±2^a^	67±7^b^	47±6^b^	32±4^a^
	1×10^5^	80±3^a^	60±7^b^	48±4^b^	31±4^a^
	1×10^7^	80±3^a^	41±5^a^	32±5^a^	26±2^a^

The standard deviation is added (mean±SD) for each value. All treatments were done in triplicate. Values in a single column for each experiment showing the same superscript letter are not significantly different (*P>*0.05).

**Table 3:** Average survival (%) of heat shocked *L. vannamei* post-larvae after challenge for 24, 48 and 72 h with 1×10^7^ *V. harveyi*/ml.

NLHS	Survival (%)		
	24 h	48 h	72 h
Control-28°C	66±2^a^	37±3^a^	23±3^a^
30°C	59±8^a^	38±5^a^	23±3^a^
32°C	58±3^a^	38±4^a^	24±2^a^
34°C	59±5^a^	38±4^a^	22±2^a^
36°C	63±3^a^	47±3^a^	28±2^a^
38°C	67±7^a^	38±4^a^	29±4^a^

The standard deviation (mean±SD) is given for each value. All treatments were performed in triplicate. Values in a single column showing the same superscript letter are not significantly different (*P>*0.05).

## Discussion

A 30 min NLHS significantly increased the amount of proPO and hemocyanin mRNA in *L. vannamei* post-larvae, in line with the observation that the shrimp immune response is influenced by temperature [5]. In contrast to the results shown here with *L. vannamei*, proPO and hemocyanin mRNA in *P. monodon* decrease significantly after 24 h heat shock [36]. The contrasting syntheses of proPO and hemocyanin mRNAs between these species may be due to the intensity of heat shock and the shrimp stages employed in the experiments.

The amount of peroxinectin mRNA in *L. vannamei* post-larvae was unchanged by NLHS, an observation similar to those described for adult *L. vannamei*. As one example, heating from 26°C to 34°C failed to induce peroxinectin production in adult *L. vannamei* within 24 h but a significant reduction occurred 2 days after heat shock, indicating that hyperthermic stress results in the loss of this antimicrobial peptide during prolonged post-stress recovery [37]. High levels of peroxinectin occur naturally in early larval stages of shrimp [38] and reduction of this immune protein may increase risk upon exposure to invading pathogens because the primary functions of peroxinectin are to regulate granule exocytosis [8], promote encapsulation [39] and mediate non-self recognition during microbial attack [40], [41].

NLHS did not alter crustin mRNA in *L. vannamei* post-larvae, this differing from observations made for other crustaceans encountering hyperthermic stress. As a case in point, transcription of the crustin*Pm5* gene is greatly increased in *P. monodon* at 35°C whereas the level was unchanged in shrimp maintained at 30°C [31]. Carcinin, a crustin-type antimicrobial protein of *Carcinus maenas* was up-regulated 2.9 fold when crabs reared at 10°C were transferred to 20°C for 2 weeks [42]. The levels of induced crustin depend on the intensity and duration of heat shock, and induction may be species-specific, especially when considering the range of physiological diversity across crustaceans. In *L. vannamei*, crustin occurs naturally in hemocytes, lymphoid organ, gill, hepatopancreas, stomach, midgut and the neural ganglion [43] and it protects against fungi [44] and several Gram positive and negative bacteria, including *Staphylococcus aureus*, *Streptococcus iniae*, *Escherichia coli* 363 and *V. harveyi* [45], [46].

Penaeidin mRNA was down-regulated in *L. vannamei* post-larvae after NLHS. The appearance of penaeidin following microbial challenge has been described but this study is the first to report the status of penaeidin after heat perturbation. Penaeidin is expressed in the hematopoietic nodules and testis of *L. vannamei*, but not in the brain, hemocyte, lymphoid organ, gill, hepatopancreas, stomach, midgut and neural ganglion after heat shock [45], [47]. Penaeidins are potent immune proteins, acting against Gram positive bacteria and filamentous fungi [17], [48]. Penaeidins are highly up-regulated after *V. harveyi* challenge, indicating these antimicrobial peptides have immunomodulatory roles against this pathogen [49]. The study described herein illustrates that protection of *L. vannamei* post-larvae against *V. harveyi* is not enhanced following proPO and hemocyanin mRNA accumulation. However, it is necessary to quantify the corresponding immune proteins to definitively determine their role in the immune status of post-larvae and tolerance to bacterial infection.

NLHS induced Hsp70 production in *L. vannamei* post-larvae, with RT-qPCR and ELISA revealing increases in mRNA and protein respectively. In juvenile *L. vannamei* 6 h heat shock from 15°C to 28°C enhanced Hsp70 mRNA accumulation 2-fold as compared to non-heated controls [50], indicating that Hsp70 synthesis across *L. vannamei* life stages is promoted by heat. Hyperthermic stress increases Hsp70 accretion in shrimp other than *L. vannamei* with 90 min heat shock from 28°C to 35°C up-regulating Hsp70 mRNA in juveniles of *P. monodon* 24-fold [51]. In another example, 30 min transfer from 28°C to 37°C with recovery at 28°C for 6 h enhanced Hsp70 build-up in larvae and adults of the brine shrimp *Artemia* 2 to 3-fold more than non-heated controls [2], [34]. These studies demonstrate that an acute hyperthermic stress triggers Hsp70 accumulation and protects shrimp against abiotic and biotic stresses.

A 30 min NLHS enhanced *Artemia* survival 2-fold against pathogenic *V. campbellii* challenge. In another example, NLHS increased *P. monodon* tolerance to Gill associated virus (GAV) [3]. In both studies, augmentation of survival following microbial challenge correlated with Hsp70 accumulation. Reduction of bacterial and viral load in hosts suggests a role for Hsp70 in pathogen attenuation [52]. NLHS however did not enhance *L. vannamei* tolerance against *V. harveyi* even though Hsp70 was significantly up regulated, indicating that protection is not afforded solely by endogenous Hsp70 accumulation as demonstrated in *Artemia* sp. [2] and *P. monodon* [3]. An increase in Hsp70 following NLHS fails to safeguard platyfish *Xiphophorus maculates* against *Yersinia ruckeri* infection, but protection is enhanced when NLHS is combined with injection of GroEL and DnaK, bacterial Hsps equivalent to Hsp60 and Hsp70 [53]. How endogenous and exogenous Hsps influence the disease tolerance of shrimp is unknown, although based on evidence for other crustaceans [54], [55], [56], immune stimulation is possible.

Elucidating associations between Hsps, the immune system and pathogen resistance in shrimp represents an intriguing challenge. It will be especially interesting to dissect this complex and highly integrated relationship in order to revisit questions regarding Hsp functions, and gain new insights into Hsp activity in commercially important aquaculture organisms such as *L. vannamei.*

## References

[1] Roberts RJ, Agius C, Saliba C, Bossier P, Sung YY (2010) Heat Shock Proteins (Chaperones) in Fishes and Shellfishes and their Potential Role in Health and Welfare: A Review. J Fish Dis 33: 789–801. 20678104 10.1111/j.1365-2761.2010.01183.x

[2] Sung YY, Van Damme EJM, Sorgeloos P, Bossier P (2007) Non-lethal heat shock protects gnotobiotic *Artemia franciscana* larvae against virulent Vibrios. Fish Shellfish Immunol 22: 318–326. 17123831 10.1016/j.fsi.2006.05.008

[3] de la Vega E, Hall MR, Degnan BM, Wilson KJ (2006) Short-term hyperthermic treatment of *Penaeus monodon* increases expression of heat shock protein 70 (HSP70) and reduces replication of gill associated virus (GAV). Aquaculture 253: 1–4.

[4] Sung YY, MacRae TH, Sorgeloos P, Bossier P (2011) Stress response for disease control in aquaculture. Rev Aquacult 3: 120–137.

[5] Pan LQ, Hu FW, Jing FT, Liu HJ (2008) The effect of different acclimation temperatures on the prophenoloxidase system and other defence parameters in *Litopenaeus vannamei* . Fish Shellfish Immunol 25: 137–142. 18455435 10.1016/j.fsi.2008.03.016

[6] Unestam T, Söderhäll K (1977) Soluble fragments from fungal cell walls elicit defence reactions in crayfish. Nature 267: 45–46. 404572 10.1038/267045a0

[7] Gillespie JP, Kanost MR, Trenczek T (1997) Biological mediators of insect immunity. Annu Rev Entomol 42: 611–643. 9017902 10.1146/annurev.ento.42.1.611

[8] Sricharoen S, Kim JJ, Tunkijjanukij S, Soderhall I (2005) Exocytosis and proteomic analysis of the vesicle content of granular hemocytes from a crayfish. Dev Comp Immunol 29: 1017–1031. 15975654 10.1016/j.dci.2005.03.010

[9] Johansson MW, Lind MI, Holmblad T, Thörnqvist PO, Söderhäll K (1995) Peroxinectin, a novel cell adhesion protein from crayfish blood. Biochem Biophys Res Commun 22: 1079–1087. 10.1006/bbrc.1995.2731 7488183

[10] Thörnqvist PO, Johansson MW, Söderhäll K (1994) Opsonic activity of cell adhesion proteins and beta-1,3-glucan binding proteins from two crustaceans. Dev Comp Immunol 18: 3–12. 8050614 10.1016/0145-305x(94)90247-x

[11] Liu CH, Cheng W, Chen JC (2005) The peroxinectin of white shrimp *Litopenaeus vannamei* is synthesised in the semi-granular and granular cells, and its transcription is up-regulated with *Vibrio alginolyticus* infection. Fish Shellfish Immunol 18: 431–444. 15683919 10.1016/j.fsi.2004.10.005

[12] Burge EJ, Burnett LE, Burnett KG (2009) Time-course analysis of peroxinectin mRNA in the shrimp *Litopenaeus vannamei* after challenge with *Vibrio campbellii* . Fish Shellfish Immunol 27: 603–609. 19490940 10.1016/j.fsi.2009.05.012

[13] Destoumieux-Garzón D, Bulet P, Loew D, Van Dorsselaer A, Rodriguez J, et al (1997) Penaeidins, a new family of antimicrobial peptides isolated from the shrimp *Penaeus vannamei* (Decapoda). J Biol Chem 272: 28398–28406. 9353298 10.1074/jbc.272.45.28398

[14] Cuthbertson BJ, Shepard EF, Chapman RW, Gross PS (2002) Diversity of the penaeidin antimicrobial peptides in two shrimp species. Immunogenet 54: 442–445. 10.1007/s00251-002-0487-z 12242595

[15] Chen JY, Pan CY, Kuo CM (2004) cDNA sequence encoding an 11.5-kDa antimicrobial peptide of the shrimp *Penaeus monodon* . Fish Shellfish Immunol 16: 659–664. 15110339 10.1016/j.fsi.2003.10.006

[16] Bachere E, Destoumieux-Garzón D, Bulet P (2000) Penaeidins, antimicrobial peptides of shrimp: a comparison with other effectors of innate immunity. Aquaculture 191: 71–88.

[17] Destoumieux-Garzón D, Bulet P, Strub JM, Van Dorsselaer A, Bachere E (1999) Recombinant expression and range of activity of penaeidins, antimicrobial peptides from penaeid shrimp. Eur J Biochem 266: 335–346. 10561573 10.1046/j.1432-1327.1999.00855.x

[18] Robertson PAW, Calderon J, Carrera L, Stark JR, Zherdmant M, et al (1998) Experimental *Vibrio harveyi* infections in *Penaeus vannamei* larvae. Dis Aquat Organ 32: 151–155.

[19] Tassanakajon A, Klinbunga S, Paunglarp N, Rimphanitchayakit V, Udomkit A, et al (2006) *Penaeus monodon* gene discovery project: the generation of an EST collection and establishment of a database. Gene 384: 104–112. 16945489 10.1016/j.gene.2006.07.012

[20] Adachi K, Hirata T, Nagai K, Sakaguchi M (2001) Hemocyanin a most likely inducer of black spots in Kuruma prawn *Penaeus japonicus* during storage. J Food Sci 66: 1130–1136.

[21] Paul RJ, Pirow R (1998) The physiological significance of respiratory proteins in invertebrates. J Zool 100: 319–327.

[22] Jaenicke E, Foll R, Decker H (1999) Spider hemocyanin binds ecdysone and 20-OH-ecdysone. J Biol Chem 274: 34267–34271. 10567401 10.1074/jbc.274.48.34267

[23] Lee SY, Soderhall K (2002) Early events in crustacean innate immunity. Fish Shellfish Immunol 12: 421–437. 12194453 10.1006/fsim.2002.0420

[24] Destoumieux-Garzón D, Saulnier D, Garnier J, Jouffrey C, Bulet P, et al (2001) Crustacean Immunity. Antifungal peptides are generated from the C terminus of shrimp hemocyanin in response to microbial challenge. J Biol Chem 276: 47070–47077. 11598107 10.1074/jbc.M103817200

[25] Zhang X, Huang C, Qin Q (2004) Antiviral properties of haemocyanin isolated from shrimp *Penaeus monodon* . J Antivir Res 61: 93–99. 10.1016/j.antiviral.2003.08.019 14670582

[26] Smith VJ, Fernandes JM, Kemp GD, Hauton C (2008) Crustins: enigmatic WAP domain-containing antibacterial proteins from crustaceans. Dev Comp Immunol 32: 758–772. 18222540 10.1016/j.dci.2007.12.002

[27] Tassanakajon A, Somboonwiwat K (2011) Antimicrobial peptides from the black tiger shrimp *Penaeus monodon* - A review, In: Bondad-Reantaso MG, Jones JB, Corsin F, Aoki T, editors. Diseases in Asian Aquaculture VII. Fish Health Section, Asian Fisheries Society, Selangor, Malaysia. 229–240.

[28] Supungul P, Tang S, Maneeruttanarungroj C, Rimphanitchayakit V, Hirono I, et al (2008) Cloning, expression and antimicrobial activity of crustin*Pm*1, a major isoform of crustin, from the black tiger shrimp *Penaeus monodon* . Dev Comp Immunol 32: 61–70. 17573111 10.1016/j.dci.2007.04.004

[29] Zhang J, Li F, Wang Z, Xiang J (2007) Cloning and recombinant expression of a crustin-like gene from Chinese shrimp, *Fenneropenaeus chinensis* . J Biotechnol 127: 605–614. 16987562 10.1016/j.jbiotec.2006.08.013

[30] Tassanakajon A, Vatanavicharn, Supungul P, Tang Sureerat, Amparyup P, et al. (2008) Biotechnology of marine invertebrates - Recent advances in shrimp and shellfish. Fisheries for Global Welfare Environment, 5^th^ World Fisheries Congress, 221–239.

[31] Vatanavicharn T, Supungul P, Puanglarp N, Yingvilasprasert W, Tassanakajon A (2009) Genomic structure, expression pattern and functional characterization of crustinPm5, a unique isoform of crustin from *Penaeus monodon* . Comp Biochem Physiol B: Biochem Mol Biol 153: 244–252. 19306939 10.1016/j.cbpb.2009.03.004

[32] Livak JK, Davey ST (2001) Analysis of relative gene expression data using realtime quantitative PCR and the 2^−ΔΔCt^ method. Method 25: 402–408. 10.1006/meth.2001.1262 11846609

[33] Sung YY, Roberts RJ, Bossier P (2012) Enhancement of Hsp70 synthesis protects common carp *Cyprinus carpio* L. against lethal ammonia toxicity. J Fish Dis 35: 563–568. 22724455 10.1111/j.1365-2761.2012.01397.x

[34] Clegg JS, Jackson SA, Hoa NV, Sorgeloos P (2000) Thermal resistance, developmental rate and heat shock proteins in *Artemia franciscana*, from San Francisco Bay and Southern Vietnam. Biochem Biophys Res Commun 252: 85–96. 10.1016/s0022-0981(00)00239-2 10962067

[35] Sung YY, Pineda C, MacRae TH, Sorgeloos P, Bossier P (2008) Exposure of gnotobiotic *Artemia franciscana* larvae to abiotic stress increases heat shock protein 70 synthesis and enhances resistance to pathogenic *Vibrio campbellii* . Cell Stress Chaperones 13: 59–66. 18347942 10.1007/s12192-008-0011-y PMC2666215

[36] de la Vega E, Bernard MD, Michael RH, Kate JW (2007) Differential expression of immune-related genes and transposable elements in black tiger shrimp (*Penaeus monodon*) exposed to a range of environmental stressors. Fish Shellfish Immunol 23: 1072–1088. 17613247 10.1016/j.fsi.2007.05.001

[37] Liu CH, Cheng W, Kuo CM, Chen JC (2004) Molecular cloning and characterisation of a cell adhesion molecule, peroxinectin from the white shrimp *Litopenaeus vannamei* . Fish Shellfish Immunol 17: 13–26. 15145414 10.1016/j.fsi.2003.11.002

[38] Jiravanichpaisal P, Pusnglsrp N, Petkon S, Donnuea S, Soderhall I, et al (2007) Expression of immune-related genes in larval stages of giant tiger shrimp, *Penaeus monodon* . Fish Shellfish Immunol 23: 815–824. 17490892 10.1016/j.fsi.2007.03.003

[39] Kobayashi M, Johansson MW, Söderhäll K (1990) The 76 kD cell adhesion factor from crayfish haemocytes promotes encapsulation in vitro. J Cell Tiss Res 260: 13–18.

[40] Holmblad T, Söderhäll K (1999) Cell adhesion molecules and antioxidative enzymes in a crustacean, possible role in immunity. Aquaculture 172: 23–111.

[41] Saulnier D, Haffner P, Goarant C, Levy P, Ansquer D (2000) Experimental infection models for shrimp vibriosis studies: A review. Aquaculture 191: 133–144.

[42] Virginia B, Valerie JS (2008) Crustin expression following bacterial injection and temperature change in the shore crab, *Carcinus maenas* . Dev Comp Immunol 32: 1027–1033. 18343497 10.1016/j.dci.2008.02.002

[43] Wang YC, Chang PS, Chen HY (2007) Tissue expressions of nine genes important to immune defence of the Pacific white shrimp *Litopenaeus vannamei* . Fish Shellfish Immunol 23: 1161–1177. 17964809 10.1016/j.fsi.2007.04.004

[44] Amparyup P, Donpudsa S, Tassanakajon A (2008) Shrimp single WAP domain (SWD)-containing protein exhibits proteinase inhibitory and antimicrobial activities. Dev Comp Immunol 32: 1497–1509. 18602420 10.1016/j.dci.2008.06.005

[45] Zhang J, Li F, Wang Z, Xiang J (2007) Cloning and recombinant expression of a crustin-like gene from Chinese shrimp *Fenneropenaeus chinensis* . J Biotechnol 127: 605–614. 16987562 10.1016/j.jbiotec.2006.08.013

[46] Supungul P, Tang S, Maneeruttanarungroj C, Rimphanitchayakit V, Hirono I, et al (2008) Cloning, expression, and anti-microbial activity of *crustinPm1*, a major isoform of crustin, from the black tiger shrimp *Penaeus monodon* . Dev Comp Immunol 32: 61–70. 17573111 10.1016/j.dci.2007.04.004

[47] Chiou TT, Lu JK, Wu JL, Chen TT, Ko CF, et al (2006) Expression and characterization of tiger shrimp *Penaeus monodon* penaeidin (mopenaeidin) various tissues, during early embryonic development and moulting stages. Dev Comp Immunol 31: 132–142. 16820207 10.1016/j.dci.2006.05.007

[48] Robertson PAW, Calderon J, Carrera L, Stark JR, Zherdmant M, et al (1998) Experimental *Vibrio harveyi* infections in *Penaeus vannamei* larvae. J Dis Aquat Org 32: 151–155.

[49] Li CY, Song YL (2010) Proline-rich domain of penaeidin molecule exhibits autocrine feature by attracting penaeidin-positive granulocytes toward the wound-induced inflammatory site. Fish Shellfish Immunol 29: 1044–1052. 20816808 10.1016/j.fsi.2010.08.020

[50] Zhou J, Wang L, Xin Y, Wang WN, He WY, et al (2010) Effect of temperature on antioxidant enzyme gene expression and stress protein response in white shrimp, *Litopenaeus vannamei* . J Thermal Biol 35: 284–289.

[51] Wanilada R, Rungnapa L, Pikul J (2010) Expression and distribution of three heat shock protein genes under heat shock stress and under exposure to *Vibrio harveyi* in *Penaeus monodon* . Dev Comp Immunol 10: 1082–1089. 10.1016/j.dci.2010.05.012 20561967

[52] Sung YY, MacRae TH (2011) Heat shock proteins and disease control in aquatic organisms. J Aquacult Res Dev S2: 006 doi:10.4172/2155-9546.S2-006

[53] Ryckaert J, Pasmans F, Tobback E, Duchateau L, Decostere A, et al (2010) Heat shock proteins protect platyfish (*Xiphophorus maculatus*) from *Yersinia ruckeri* induced mortality. Fish Shellfish Immunol 28: 228–231. 19751832 10.1016/j.fsi.2009.09.005

[54] Sung YY, Dhaene T, Defoirdt T, Boon N, MacRae TH, et al (2009) Ingestion of bacteria over-producing DnaK attenuates *Vibrio* infection of gnotobiotic *Artemia franciscana* larvae. Cell Stress Chaperones 14: 603–609. 19373565 10.1007/s12192-009-0112-2 PMC2866948

[55] Sung YY, Ashame MF, Chen SJ, MacRae TH, Sorgeloos P, et al (2009) Feeding *Artemia franciscana* (Kellogg) larvae with bacterial heat shock protein, protects from *Vibrio campbellii* (Baumann) infection. J Fish Dis 32: 675–685. 19515074 10.1111/j.1365-2761.2009.01046.x

[56] Baruah K, Ranjan J, Sorgeloos P, MacRae TH, Bossier P (2011) Priming the prophenoloxidase system of *Artemia franciscana* by heat shock proteins protects against *Vibrio campbellii* challenge. Fish Shellfish Immunol 31: 134–141. 21554959 10.1016/j.fsi.2011.04.008

